# A note on the the discovery of Chagas' disease

**DOI:** 10.1590/0074-02760210372chgsb

**Published:** 2022-05-16

**Authors:** Fernando Dias de Avila-Pires

**Affiliations:** 1 Fundação Oswaldo Cruz-Fiocruz, Instituto Oswaldo Cruz, Departamento de Medicina Tropical, Rio de Janeiro, RJ, Brasil

The article lives up to the authors’ expertise and reputation and it is an honor to be invited to join in this panel. I’m going to highlight some outstanding points and raise a few questions for consideration by the readers.

Statements in *italics* when not stated otherwise are quotes from Simone and Nísia’ paper.

*Knowing that blood-feeding insects in buildings can be vectors of parasitic diseases, the scientist examined the bugs and identified T. cruzi.*

Carlos Justiniano Ribeiro das Chagas has been praised as the single parasitologist who discovered a parasite, its vector, its reservoirs and the disease it causes. As Coutinho and Dias wrote:

*Chagas orquestrou um excepcional discurso de descoberta, contemplando a biologia do parasita, seu ciclo de vida e seu modo de transmissão. Além disso, ele produziu uma descrição clínica da nova doença*.^1^

*Individual action is inarguably a vital dimension of history and social life. However, historians and social scientists have come to insist that science, like other forms of knowledge production, is essentially a collective activity, one not realised by isolated individuals but by concrete communities and groups that share ideas, practices, procedures, convictions, values, and spaces.*

Nowadays science, like history, is considered to be a collective undertaking. A scientist toiling alone in the far reaches of Lassance, at the dawn of the 20 th century is a relic of the past. Indeed, the invention of a new vaccine, a new drug and the achievement of a lunar exploration require the collaboration of a group of scientists working in harmony.

However, if science is arguably a collective activity, why is the Nobel Prize awarded to a single individual? When it is attributed to more than one the recipients, in most cases work for different institutions and in different countries. In fact, if the work is usually collective in certain fields, the creativity of the scientist, the painter and the composer remains a lonely endeavor.

*In contrast with a long-enduring tradition of epistemologists who contended that scientific theories and facts should be analysed within the pure domain of methodological procedures by which they could be verified experimentally*. […] *Field sciences were long seen as a preliminary stage of research, epistemologically secondary to the type of science practiced in the laboratory*. […] *that is, the field was considered a place expressly dedicated to collecting raw material for examination and transforming it into knowledge under controlled laboratory conditions.*

A statement that deserves a longer discussion concerns the old issue of *field sciences* and *observational studies* versus *experimental* sciences.^2^

Actually raw data may be acquired in the lab as well. In the field as astronomy, paleontology, systematics, and in several others, experimentation is hardly feasible.

In medicine, the laboratory entered the hospital only after Pasteur.^3^ As a curiosity, according to the Hipocratic theories concerning the influence of *waters*, *airs*, and *places* on the incidence of diseases, the 1862 Report of the Faculty of Medicine of Rio de Janeiro announced the installation of a *meteorological station* in the Sessions Room because there was no room for it in the Hospital Santa Casa da Misericordia.

*The new illness controversy waged inside the National Academy of Medicine (Academia Nacional de Medicina) in 1922 and 1923* […]. ***When Afrânio Peixoto classified American trypanosomiasis as a mere “illness from Lassance”, he was challenging both the position that Chagas disease had attained as an emblem of Brazilian biomedical research as well as the political meanings ascribed to it by the rural sanitation movement. In Peixoto’s view, the idea of a country beset by tropical illnesses fed into longstanding European biases about “the backwards tropics” and damaged the country’s image abroad, frightening off capital and immigrants*** .

The bitter discussions in the Academy of Medicine argued the importance and the geographic distribution of the new disease throughout Brazil’s hinterland and its political implications, not its identity.

*The scientific factors pertained to valid research questions concerning some of Chagas’s statements about the clinical presentation of the disease, especially about the correlation between endemic goiter and T. cruzi infection.*

The authors rightly pointed out the most relevant problematical aspect of the issue, the one concerning the identity of the disease that Chagas described in 1909.

This question was aptly raised and answered by the French historian and sociologist François Delaporte in his book *La maladie de Chagas*.^4^

The disease described by Chagas in 1909 was a chimera, denominated *parotidite parasitaria* or *parasitic goiter*. Chagas combined the symptoms of two distinct pathological entities, endemic goiter and what would be appropriately re-described as *American trypanosomiasis*.

The description of a composite animal species is a frequent issue in zoological taxonomy. The International Code of Zoological Nomenclature tackles a similar problem. When an author describes a new species and a succeeding taxonomist shows that the preceding author failed in recognising that his “species” is actually a composite of two distinct taxons he must describe a new species and preserve the previous species name and original author for the original species, with the suffix *partim*.

In the case of Chagas disease, Carlos Chagas described in 1909 a composite disease he named parasitic goiter. He actually described a new parasite, its natural host, and the signals and symptoms of a cardiopathy. But the new disease was a composite of two distinct entities.

*The disease itself was thus to frame a project for the society and the nation, one that found expression in Chagas’s engagement in the rural sanitation movement, which brought together scientists, intellectuals, and politicians to call for the Brazilian State to implement public policies that would serve the ailing people of the country’s interior.*

Chagas rightly recognised the importance of a new disease for the future of public health as the authors of the article aptly recognised.

It would be up to one of our most notable nationalist writers, José Bento Renato Monteiro Lobato (April 18, 1882 - July 4, 1948) to show, in its stark reality, the misery of our hinterland through the fictional character of the novel *Jeca Tatu* who had been rendered non-productive by the ancilostom parasitic disease.

A final comment on an important issue, one that exemplifies the interweaving aspects of the cognitive and social aspects of science was the controversy between those who advocated and those who rebuffed Tropical Medicine as a specialty in its own right. It will be up to the readers to judge for themselves.

In its original sense Tropical medicine was thus described by Manson in the introduction to his volume *Tropical diseases: a manual of the diseases of warm climates* ^5^ in 1898:

*The title which I have elected to give to this work, Tropical Diseases, is more convenient than accurate*. […]. *I employ the term “tropical” in a meteteorolical rather in a geographical sense, meaning by it sustained high atmospheric temperature; and by the term “tropical diseases” I wish to indicate diseases occurring only, or which from one circumstance or another are especially prevalent, in warm climates.*

If not scientifically accurate, the concept is convenient and useful.

As an Appendix I suggest the article on *Ensino de pós-graduação em medicina tropical*, a conference delivered by José Rodrigues Coura during the X Congress of the Brazilian Society of Tropical Medicine, Curitiba, February 3-6, 1974, available in https://www.scielo.br/scielo.php?script=sci_arttext&pid=S0037-86821974000100001&lng=pt&tlng=pt.

Comments on the article: Kropf SP, Lima NT. The history of Chagas disease: reflections on science in action. Mem Inst Oswaldo Cruz. 2022; 117: e200372.
